# A Comparative Analysis of Speed Profile Models for Ankle Pointing Movements: Evidence that Lower and Upper Extremity Discrete Movements are Controlled by a Single Invariant Strategy

**DOI:** 10.3389/fnhum.2014.00962

**Published:** 2014-11-27

**Authors:** Konstantinos P. Michmizos, Lev Vaisman, Hermano Igo Krebs

**Affiliations:** ^1^Martinos Center for Biomedical Imaging, Massachusetts Institute of Technology, Massachusetts General Hospital, Harvard Medical School, Charlestown, MA, USA; ^2^McGovern Institute for Brain Research, Massachusetts Institute of Technology, Cambridge, MA, USA; ^3^Department of Anatomy and Neurobiology, School of Medicine, Boston University, Boston, MA, USA; ^4^Department of Mechanical Engineering, Massachusetts Institute of Technology, Cambridge, MA, USA; ^5^Department of Neurology, Division of Rehabilitation, School of Medicine, University of Maryland, College Park, MD, USA; ^6^Department of Physical Medicine and Rehabilitation, Fujita Health University, Nagoya, Japan; ^7^Institute of Neuroscience, University of Newcastle, Newcastle upon Tyne, UK

**Keywords:** sensorimotor control, ankle movements, rehabilitation robotics, neurorehabilitation of motor function, stroke, cerebral palsy

## Abstract

Little is known about whether our knowledge of how the central nervous system controls the upper extremities (UE), can generalize, and to what extent to the lower limbs. Our continuous efforts to design the ideal adaptive robotic therapy for the lower limbs of stroke patients and children with cerebral palsy highlighted the importance of analyzing and modeling the kinematics of the lower limbs, in general, and those of the ankle joints, in particular. We recruited 15 young healthy adults that performed in total 1,386 visually evoked, visually guided, and target-directed discrete pointing movements with their ankle in dorsal–plantar and inversion–eversion directions. Using a non-linear, least-squares error-minimization procedure, we estimated the parameters for 19 models, which were initially designed to capture the dynamics of upper limb movements of various complexity. We validated our models based on their ability to reconstruct the experimental data. Our results suggest a remarkable similarity between the top-performing models that described the speed profiles of ankle pointing movements and the ones previously found for the UE both during arm reaching and wrist pointing movements. Among the top performers were the support-bounded lognormal and the beta models that have a neurophysiological basis and have been successfully used in upper extremity studies with normal subjects and patients. Our findings suggest that the same model can be applied to different “human” hardware, perhaps revealing a key invariant in human motor control. These findings have a great potential to enhance our rehabilitation efforts in any population with lower extremity deficits by, for example, assessing the level of motor impairment and improvement as well as informing the design of control algorithms for therapeutic ankle robots.

## Introduction

At least for stroke, robot-assisted therapy results in substantial improvement in the upper extremities (UE), and the improvement is superior to conventional therapy (Kwakkel et al., 2008). These results led the American Heart Association to endorse in its 2010 guidelines for stroke care the use of robots for UE rehabilitation (Miller et al., 2010) followed up by a similar endorsement issued by the veterans administration (VA) later in the same year (Management of Stroke Rehabilitation Working Group, 2010). To date, though, there is no clear evidence that the same is true for the lower extremities (LE). In fact, recent studies employing the Lokomat (Hocoma, Zurich, Switzerland), the most widely used LE robotic rehabilitation device, reported inferior outcomes compared to those produced by usual care as practiced in the US for both chronic and subacute stroke patients (Hornby et al., 2008; Hidler et al., 2009). Not surprisingly, guidelines issued by the VA currently recommend against the use of LE devices post-stroke by its clinicians (Management of Stroke Rehabilitation Working Group, 2010).

The lack of superior results in LE robotic therapy could be attributed to therapeutic approaches that are limited by unverified neurological sensorimotor inputs. Since the neuroscientific paradigm shift on activity-dependent plasticity and its experimental support (Jenkins and Merzenich, 1987; Nudo et al., 1996), the sensorimotor control of UE movements has been studied extensively. Kinematic analyses and modeling of simple reaching movements have been followed up by analyses of single peak UE speed profiles (Bizzi et al., 1976; Kelso et al., 1979; Soechting and Lacquaniti, 1981; Abend et al., 1982; Flash and Hogan, 1985) and of more complex movements that were modeled as a combination of elementary movements (submovements) (Plamondon et al., 1993; Doeringer and Hogan, 1998). These and subsequent studies built an insight into how the central nervous system (CNS) controls and learns UE movements (Shadmehr and Mussa-Ivaldi, 1994; Flanagan and Rao, 1995; Bhushan and Shadmehr, 1999). In turn, these data have provided a basis for the design of manipulanda for UE therapeutic robots (Krebs et al., 1998; Volpe et al., 2000) as well as the establishment of quantitative metrics for patients’ motor recovery and performance (Krebs et al., 1999, 2014; Rohrer et al., 2002; Bosecker et al., 2010). On the other hand, the design for robotic devices for LE attempted to automate current gait therapy practices, in particular body-weight-supported treadmill training. However, this perceived “gold standard” has not been carefully tested until recently when significant poor results were observed (Duncan et al., 2011; Dobkin and Duncan, 2012).

There is a scarcity of studies addressing the question of whether the sensorimotor control of the LE resembles, and to what extent, that of the UE. Functionally, the LE are specialized to support body weight, provide locomotion, and maintain stability, whereas the UE are better suited for intrinsic mobility and dexterity (Netter, 2014). This difference between working (UE) and walking (LE) our way through the world comes alongside differences in the size of the limbs, supporting muscles, articulating joints, tendons, and ligaments. Specifically, the weight of the structures located superiorly to the sacrum is supported by the pelvic girdle, a ring formed by the hip bones and the sacrum. The extensive fusion within the pelvic girdle and the greater stability of the LE joints are adaptations for weight bearing, which come at a cost of less dexterity and mobility than the UE (Waugh and Grant, 2014). Our recent studies on the sensorimotor control of the ankle revealed interesting similarities and important differences between the LE and the UE. We investigated the trade-off between speed and accuracy in goal-directed pointing movements with the ankle in dorsal–plantar (DP) and inversion–eversion (IE) and found that a linear model, widely used to quantify the UE motor system for more than half a century (Fitts’ law), also applies to the ankle (Michmizos and Krebs, 2014b). In addition to the macroscopic assessment of the average ankle speed, a microscopic study on reaction time (RT) revealed that ankle RT increases as a linear function of potential target stimuli, as would be predicted by Hick–Hyman law in UE. Interesting enough, the intercept in the regression is significantly smaller in DP than in IE direction; this could be attributed to differences in the cognitive components, including motor preparation and execution, that affect RT (Michmizos and Krebs, 2014a,c).

We became interested in the ankle because of its crucial role in human walking and because a deficit in foot control is the most common and debilitating condition in upper motor neuron disorders involving the corticospinal tract, such as stroke and cerebral palsy (CP). The ankle contributes to the maintenance of stable upright posture in the frontal and sagittal planes during gait and to shock absorption during locomotion by attenuating the impact force at floor contact (Bahlsen and Nigg, 1987). The ankle muscles are the primary contributors to overground gait – the soleus is the propulsion prime-mover, the gastrocnemius is the posture prime-mover, and the tibialis anterior (TA) is critical for toe-off (McGowan et al., 2008). In the LE, a common condition that occurs in stroke and CP is weakness in the dorsiflexor muscles that lift the foot during walking, commonly referred to as “drop foot.” The two major complications of drop foot – slapping of the foot after heel strike (foot slap) and dragging of the toe during swing (toe drag) – present a major challenge to efficient gait since clearing the ground during the swing phase and maintaining ankle stability during the stance phase are essential for efficient gait. With 800,000 Americans experiencing a new or recurrent stroke each year (Go et al., 2014), graying of the population with consequent increase in the number of Americans having a stroke each year, and CP affecting 1 to 4 per 1,000 live births worldwide (Odding et al., 2006), there is a greater need now than ever before for LE robotic rehabilitation to realize its promises.

Given the importance of active participation during therapy and the need for specificity in therapeutic tasks that resemble (if not exploit) motor learning, we are translating the concept of adaptive assist-as-needed robotic therapy, introduced for the UE (Krebs et al., 2003), to the needs and special characteristics of the LE (Michmizos and Krebs, 2012a). Briefly, our algorithm identifies the ability of the patient to move and point with the ankle in visually guided, visually evoked games (Michmizos and Krebs, 2012b), and then independently adjusts the speed of the gameplay and the size of the target not only to track patients abilities but also to challenge them (Michmizos and Krebs, 2012a). Our performance-based adaptive games have embedded into their design the assumption that ankle movements typically follow a minimum jerk profile (Flash and Hogan, 1985; Michmizos and Krebs, 2012a). Nevertheless, to our knowledge, no study has ever modeled the kinematics of ankle pointing movements. The lack of descriptive models for ankle pointing movement limits the validity of our efforts on designing an ideal therapeutic intervention. To overcome this pitfall, we experimented on whether the CNS has developed individual control strategies and kinematic features for the ankle control. Establishing a model-based analysis for human ankle movements could inform the use of metrics that quantify the level of motor-related impairment, at least in the ankle, and provide targeted therapy tailored to the patient’s inability to move the LE.

The goal of this study was to test a multitude of existing kinematic models, initially developed to describe simple UE movements, and find the ones, if any, that were the most competent in describing ankle pointing movements or else build a model best suited to the ankle. In addition to being a different modality, the ankle presents a second fundamental constraint for our modeling purposes: ankle and wrist movements are defined as finite spatial rotations (Pretterklieber, 1999; Charles and Hogan, 2011; Vaisman et al., 2013), which do not form a vector space as reaching arm movements do. To allow for a straight comparison, we selected the same 19 models from the comparative studies by Plamondon et al. (1993) and Stein et al. (1988) that we used in reaching movements and also in our wrist modeling study (Vaisman et al., 2013). Employing our adult anklebot (Roy et al., 2009), we recorded and analyzed the speed profiles of 1,386 fast, target-directed ankle movements. For each movement and model, we used a non-linear, least-squares optimization procedure to extract a set of parameters that minimized the error between the experimental data and the reconstructed speed profiles. Our goal was to determine whether any of the previously proposed models for UE movements simulated the ankle speed profiles well or, alternatively, whether new models should be investigated. We found that the top- and the worst-performing models, as well as their relative order, proposed by our ankle speed profile analysis were remarkably identical to the ones proposed by our wrist study.

## Materials and Methods

### Subjects

Fifteen unimpaired healthy human subjects (three females) were recruited for this study. Subjects were predominantly Caucasians and were postdoctoral, graduate, or undergraduate students at the Massachusetts Institute of Technology. Average biometric data were 26 ± 4 years of age, 1.77 ± 0.08 m in height, and 73 ± 11 kg in mass (mean ± SD). All subjects had normal or corrected-to-normal vision and were right-foot dominant according to their preferential use of the foot during daily activities, such as kicking a ball. Subjects had no reported history of traumas or neuropathies to the lower limbs. All subjects were naive to the task upon enrollment and gave written informed consent according to the procedure approved by the Massachusetts Institute of Technology Committee on the Use of Humans as Experimental Subjects.

### Apparatus

**Figure 1:**
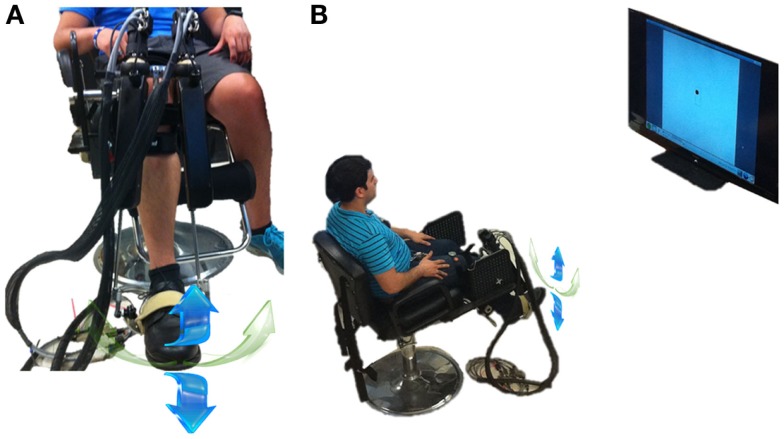
**Experimental setup**. **(A)** Subject wearing the anklebot in a seated condition. Right leg is in the anatomically neutral position. The weight of the anklebot is supported by the chair through the bolt. The DP (IE) movement direction is noted with the blue (green) arrows. Although internal-external rotation was biomechanically possible, it could not constrain the data analysis in IE direction. **(B)** Experimental setup with the subject wearing the anklebot in a seated position, facing a 60-inch monitor. From both sub-figures, irrelevant background has been removed.

The speed profiles were measured for DP and IE ankle pointing movements using a highly back-drivable wearable robot, Anklebot (Interactive Motion Technologies, Watertown, MA, USA). The robot’s design and measurement capabilities have been described previously (Roy et al., 2009). Briefly, it is a low-friction, back-drivable exoskeleton robot with intrinsically low mechanical impedance that allows normal range of motion (ROM) in all three degrees-of-freedom of the foot relative to the shank during walking overground or on a treadmill. The robot allows the maximum ROM required for the typical gait of healthy or pathological subjects (Perry, 1992) while providing independent, active assistance in DP and IE degrees-of-freedom, and a passive degree-of-freedom for internal–external rotation. The kinematic design consists of two linear actuators mounted in parallel such that if both push or pull in the same direction, a DP flexion torque is produced at the ankle. Similarly, if the two links push or pull in opposite directions, IE torque results. For this study, the anklebot acted as a passive device and recorded simultaneously the DP and IE positions. We recorded ankle position kinematics with respect to the neutral position, defined as the sole being at a right angle to the tibia. Recordings were made at 200 Hz sampling frequency and were converted to screen pixels for visualization purposes. Subjects wore a modified shoe and a knee brace to which the robot was connected. Subjects were seated, and the knee brace was securely fastened to the chair to fully support the weight of the robot and to ensure that measurements were made in a repeatable posture (Figure 1A). The chair was placed 1.5 m away from a 60-inch 1080p (Full HD) 120 Hz 1,024 × 768 Liquid Crystal TV (Sharp LC60L, Sharp Electronics Corporation) that was positioned at eye level (Figure 1B). Visual feedback was given online to the subjects as a moving circular cursor (*d* = 23 pixels). A DP (IE) movement of the ankle moved the cursor vertically (horizontally); hence, the cursor moved in a 2D coordinate system with the origin corresponding to the ankle’s neutral position. Visualization software was written in TCL/TK and run on a PC equipped with Linux Ubuntu operating system.

### Experimental procedure

Subjects were instructed to control with their ankle the cursor moving vertically (for DP motion) or horizontally (for IE motion). Participants moved the cursor between two rectangular targets “as fast and accurately as possible” by dorsi-/plantar flexing or inverting/everting their ankle. They were getting an online visual feedback of their performance by a cursor moving toward the onscreen target. Due to the visual feedback being equal for all directions, we ensured the same feedback resolution across both movements. The maximum ankle angle with respect to the neutral position was 0.2 rads (12°); it was constrained by the ankle’s biomechanics and specifically by the ROM for dorsiflexion (20°), which is the minimum across the four tested directions (Perry, 1992). When the target was in vertical (horizontal) direction, the moving cursor was constrained to vertical (horizontal) movements. The subjects first placed the cursor at the center of the screen by positioning their ankle in the neutral position. Next, a rectangular target was shown up (right) or down (left), for the DP (IE) direction, and subjects made an outbound movement, i.e., they moved their ankle away from the neutral position. Outbound targets were presented in a random order, so that the subject could not anticipate movements. For the movement to be considered as discrete, the cursor had to land inside the rectangular target (i.e., not touching any of its borders) and its instantaneous speed had to be lower than 0.001 rad/s. The first criterion ensured that all discrete movements had amplitudes with the least possible variation. The second criterion was empirically set to detect accurately discrete movements, given the sampling frequency of recording the position of the Anklebot’s linear actuators (200 Hz). When both criteria were met, the outbound target disappeared, and an inbound target, i.e., a target around the central position, appeared instructing the subject to move his/her ankle to its neutral position. Subsequently, after a dwell time randomly ranging between 800 and 1,200 ms, a new outbound target appeared. Each subject participated in one DP and one IE session each of which consisted of 180 movements. The diameter of the cursor was 0.015 rads (23 pixels) and the target width, in the direction of the movement, was randomly selected between 0.03 rads (45 pixels) and 0.08 rads (120 pixels), with a step increment of 0.01 rad (15 pixels). Note that as we were not interested in this study for the effect, if any, of the size of the target on the speed profiles, we concatenated our data across target widths for each ankle direction. Random presentation of differently sized targets and short overall duration of the experiment (<3.5 min, per direction) ensured that no fatigue would happen during a session. Although internal–external rotation during IE movements was biomechanically possible, we made certain that it did not constrain our analysis. The subjects were instructed to move their ankle in IE before data acquisition and the visual feedback ensured a consistent displacement in the IE direction (i.e., the two parallel linear actuators should be displaced in opposite directions by 0.2 rads for the cursor to land inside the target).

### Kinematic analysis and modeling

Ankle speed profiles for DP and IE movement were estimated using a simple linearized mathematical model of the shank-ankle-foot system; see Supplementary Material. After estimating the ankle angle measured from the neutral position in the sagittal (frontal) plane, θ_dp_, (θ_ie_), we processed the ankle kinematics as in our previous studies (Vaisman et al., 2013; Michmizos and Krebs, 2014b). Briefly, we first found the time point at which the maximum speed occurred, *t*_max_. We then found the starting and stop times, *t*_start_ and *t*_stop_, respectively, for each movement, by measuring the duration of the movement until its velocity fell below 5% of the maximum of speed in forward and backward directions. Next, we visually inspected the speed profiles to assure the presence of a single peak. We excluded all movements with corrections, easily identified as the ones having more than one peak with maximum amplitude at least equal to 20% of the maximum peak. On average, we selected 16 (24) out of 45 speed profiles for IE outbound (inbound) movements and 25 (29) out of 45 speed profiles for DP outbound (inbound) movements (for a total of 1,386 profiles).

To allow the speed profile segments to be of different lengths, we normalized movement duration to a range of [0, 1], as described in Bizzi et al. (1976). We subtracted *t*_start_ from all time points and then divided the resultant time point by *t*_stop_ − *t*_start_. Therefore, we did not re-sample the data and each speed profile retained its original number of time points. For each speed profile, we estimated the peak and average speed as well as its skewness and kurtosis. To evaluate the skewness and kurtosis, we treated the speed profiles as probability distribution functions (normalized so that the area under the curve was equal to 1); we then calculated $\mathrm{Skewness}=E[{\left(x-\bar{x}\right)}^{3}]∕\sigma^{3}$ and $\mathrm{Kurtosis}=E\left[E\left({\left(x-\bar{x}\right)}^{4}\right)∕\sigma^{4}\right]$, where *x* was the normalized speed profile, and $\bar{x},$ σ were its mean and standard deviation, respectively.

The equations of the models used in this study and their initialization values are listed in Supplementary Material. The optimal estimation of the model parameters was done by the interior-reflective Newton algorithm, implemented in the MATLAB function lsqcurvefit (Mathworks Inc., Natick, MA, USA), which solves a non-linear, least-squares problem (Coleman and Li, 1996). Initialization values were selected by trial and error in order to achieve convergence for each model to our data and were similar to our initialization values in our wrist study. The optimization algorithm ran for at least 100,000 iterations or until the change in squared sum of the residuals became smaller than 10^−9^. The goodness of fit for each model is reported as the percent error for the area under the fitted speed profile and the measured speed profile. The percentage is calculated as the ratio of the area under the absolute value of the residuals to the area under the speed profile curve.

To compare models with a different set of parameters, we used the Akaike information criterion (AIC). AIC values (lower is better) were estimated as AIC = *N* × ln(SSE/*N*) + 2 × *K* + 2 × *K*(*K* + 1)/(*N* − *K* − 1), where SSE is the sum of the squared residuals returned by the optimization algorithm, *N* is the number of points in the fit, and *K* is the number of model parameters plus one (Motulsky and Christopoulos, 2004). For each subject and movement direction, we estimated (a) the “Top-5” plot, where for each speed profile we awarded a model with one point when its AIC was among the 5 best performers for that profile and (b) the “Score-18” plot, where for each speed profile we ranked the models and awarded 18 points to the best performing model, 17 for the model with the second to best AIC, etc. (Vaisman et al., 2013). For each model, the resulting sum of the speed profiles was normalized by the number of profiles that contributed to the sum. While the “Top-5” plot is more specific in detecting any differences in performance among movement directions, the “Score-18” system favors consistent high level of performance and, therefore, allowed a somewhat more balanced comparison between the models.

For the statistical analysis of the modeled speed profiles, we employed the Welch analysis of variance (ANOVA) on the measures of the speed profiles properties (average and maximum speeds, skewness, and kurtosis) for different movement directions, followed by a *post hoc* multiple comparisons Games–Howell analysis to perform pairwise group comparisons (Games and Howell, 1976). This was done because the number of speed profiles in each group varied from 83 to 430, and because the variances for the groups were found to be unequal based on the Bartlett’s test for equality of variances assumption. We used the Kruskal–Wallis non-parametric one-way ANOVA test to compare the performance of the models for each group of speed profiles (Wackerly et al., 2007). The Kruskal–Wallis test was used because AIC values for most models did not satisfy the Lilliefors normality test at a 5% significance level and, therefore, AIC values could not be assumed to follow a normal distribution.

## Results

### Ankle speed profiles

**Figure 2:**
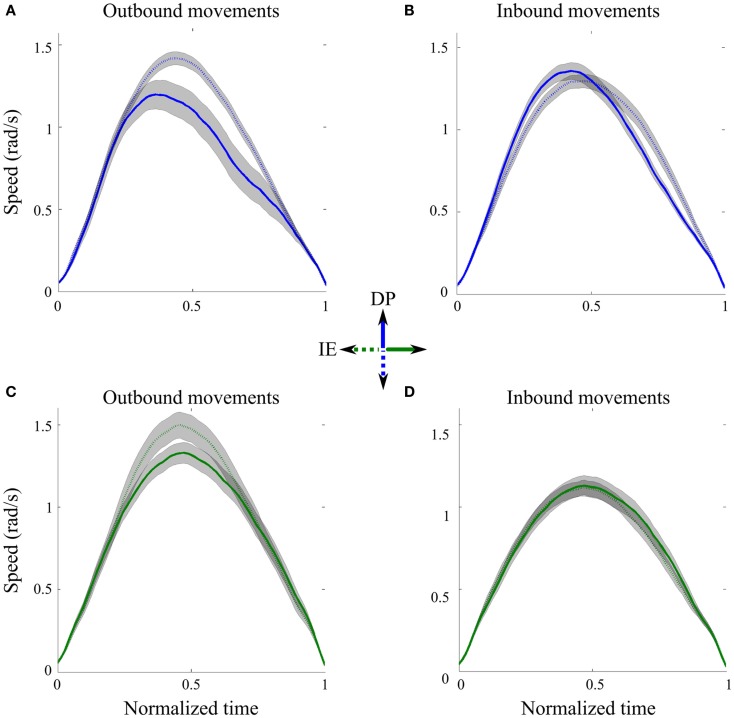
**Speed profiles across directions**. **(A)** Outbound DP pointing movements from neutral position toward north (solid line) and south (dotted line) targets, averaged across subjects. **(B)** Inbound DP pointing movements from north (solid line) and south (dotted line) targets toward the neutral position, averaged across subjects. **(C)** Outbound IE pointing movements from neutral position toward east (solid line) and west (dotted line) targets, averaged across subjects. **(D)** Inbound IE pointing movements from east (solid line) and south (dotted line) targets toward the neutral position, averaged across subjects. Gray shaped regions correspond to 95% precision uncertainty values.

Speed profiles were consistent in all directions and across subjects. Figure 2 shows the average speed profiles and their corresponding 95% precision uncertainty values for the 8 (4 outbound and 4 inbound) ankle movements. For each direction, the outbound movements of divergent orientations were different in shape when we compared their speed profiles (Figures 2A,C). Specifically the peak speeds for movements toward the south and west outbound targets were significantly bigger than those for the north and east outbound targets, respectively. On the contrary, the inbound movements of divergent orientations were similar in shape, especially when we compared the peak speed as well as the beginning and the end of the movement (Figures 2B,D); therefore, in the subsequent analysis, we concatenated all inbound IE movements and all inbound DP movements, across subjects.

### Analysis of ankle kinematics

Our *post hoc* Games–Howell analysis of the six different ankle movements revealed not only interesting similarities but also important differences across directions (Table 1). The comparison of the mean ranks between DP and IE inbound movements revealed statistical differences for all the tested features. For outbound movements, all tested features except kurtosis were found to be significant different between DP toward north and IE toward east directions. Our analysis of speed revealed that the peak and the average speeds were similar for the IE outbound, the DP inbound and the DP outbound toward south movements. Interestingly, for DP outbound to north movements, the average (but not the peak) speed was significantly lower than the other DP movements. With respect to the shape characteristics of the speed profiles, the DP outbound to north movements were more positively skewed; in addition, all movement directions had similar kurtoses with the exception of the IE inbound movements that were more platykurtic.

**Table 1:** ***Post hoc* Games–Howell pairwise comparisons for the mean ranks of the ankle speed profile features across groups (movement directions)**.

Movement direction	Speed profile feature	Mean rank	Movement direction					
			DP inbound	IE inbound	IE outbound to east	IE outbound to west	DP outbound to north	DP outbound to south
DP inbound	Max	727.5		8.7 (2.6e-4)	4.9 (7.6e-3)			
	Avg	731.4		7.1 (2.7e-4)			5.4 (3.8e-3)	
			–					
	Skew	717.5					4.8 (1.1e-2)	
	Kurt	701.1		4.1 (4.7e-2)			
IE inbound	Max	493.1			10.2 (2.6e-4)	7.6 (2.6e-4)		9.6 (2.6e-4)
	Avg	534.4			7.3 (2.6e-4)	5.9 (6.1e-4)		8.6 (2.6e-4)
			–	–				
	Skew	614.9					6.8 (7.0e-4)	
	Kurt	585.0			5.1 (4.1e-3)		4.4 (2.8e-2)	4.7 (1.1e-2)
IE outbound to east	Max	930.8					4.6 (1.4e-4)	
	Avg	887.4					6.6 (2.9e-4)	
			–	–	–			
	Skew	666.5					4.7 (7.0e-4)	
	Kurt	793.4									
IE outbound to west	Max	780.0						
	Avg	766.0					5.3 (3.0e-3)	
			–	–	–	–		
	Skew	619.7					5.8 (7.4e-4)	
	Kurt	723.8						
DP outbound to north	Max	645.1						
	Avg	481.0						6.5 (3.1e-4)
			–	–	–	–	–		
	Skew	958.9						4.7 (1.6e-2)
	Kurt	834.0						

*Max, maximum speed; Avg, average speed; Skew, skewness; Kurt, kurtosis, for each movement directions*.

*When a statistical difference was proposed by the test, the *q-*statistic (*p*-value) are reported*.

### Modeling ankle kinematics

**Figure 3:**
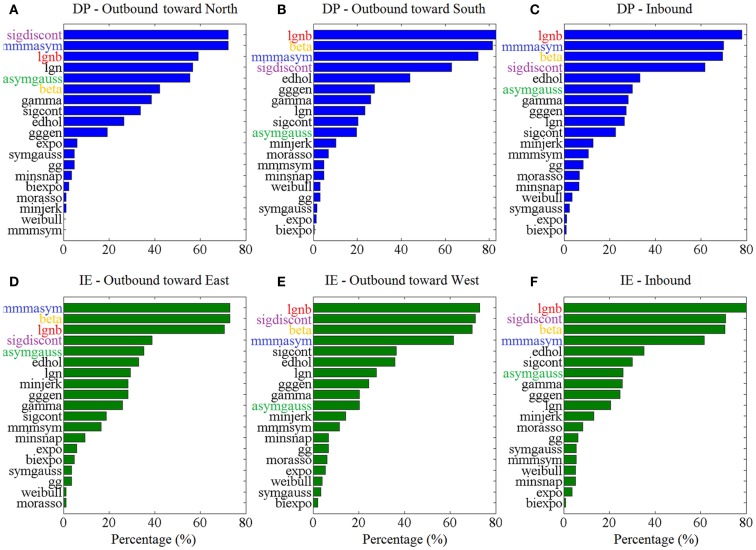
**Top – 5 plots, across ankle directions**. Percentage of speed profiles for which a model’s fit was among the 5 best fits, according to the AIC. The 5 models with the best overall performance are colored. Percentages are estimated out of **(A)** 83 outbound toward north speed profiles, **(B)** 291 outbound toward south speed profiles, **(C)** 430 DP inbound speed profiles, **(D)** 85 outbound toward east speed profiles, **(E)** 148 outbound toward west speed profiles, **(F)** 349 IE inbound speed profiles.

**Figure 4:**
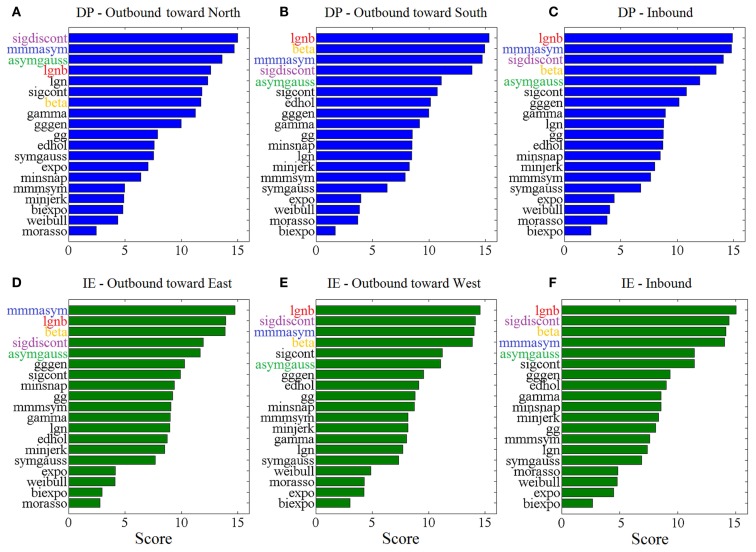
**Score – 18 plots, across ankle directions**. Higher scores (out of 18) correspond to better fits. The 5 models with the best overall performance are colored. Scores are based on **(A)** 83 outbound toward north speed profiles, **(B)** 291 outbound toward south speed profiles, **(C)** 430 DP inbound speed profiles, **(D)** 85 outbound toward east speed profiles, **(E)** 148 outbound toward west speed profiles, **(F)** 349 IE inbound speed profiles.

Figure 3 shows the “Top-5” plots across movement directions. Although the ankle speed profiles had features, such as skewness and kurtosis that were different across directions, the top-performing models had consistently the best (lowest) AIC values, irrespective of the movement direction. Specifically, the lognormal with support bound (lgnb), the Morasso Mussa-Ivaldi and Maarse asymmetric (mmmasym), the asymmetric Gaussian (asymgauss), the beta function (beta), and the sigmoidal discontinuous (sigdiscont) models outperformed all other models, across the tested ankle movement directions. A broader view of the results also revealed that, overall, the asymmetric models outperformed the symmetric models. Figure 4 shows the cumulative “Score-18” plots, across movement directions. The best performing models were the same as in the “Top-5” plot and their relative placement in the first five positions was more consistent than in Figure 3. A visual comparison of the models’ performance across the six directions revealed three abrupt performance discontinuities that we used to define three different levels of model performance. Specifically, in addition to the best performance family, there was another family of models with a performance that was consistently the worst across directions. These were the Weibull (weibull), the minimum acceleration (morasso), the biexponential (biexpo), and the exponential (expo) models. The third family of models, with an in-between performance, was composed of the remaining 10 models. A comparison between Figures 3 and 4 revealed that models like the mmmsym and minimum jerk were ranked higher in “Top-5” plots than in “Score-18” plots because, although they performed very well for some profiles ranking on the “Top-5” plot, they did not have very consistent performance for a large number of profiles and thus were ranked lower overall. The opposite was true for other models, such as the Eden and Hollerbach (edhol) and the sigmoidal continuous (sigcont) models. The models that did not perform well underperformed in both “Top-5” and “Score-18” plots.

Table 2 includes the values for the goodness of fit for all grouped speed profiles across the tested models. We compared the model fits with the wrist goodness of fit values (Vaisman et al., 2013). Overall, most models exhibit comparable error fits across modalities; note that a direct comparison is not straightforward, as the ankle data were subgrouped into six categories (movement directions), while the error fits reported in our wrist study are not. However, the better fitting models for the wrist data remain as such for the foot data, e.g., the mmmasym, the asymgauss, and the LGNB models are among the best performing of the models, in terms of goodness of fit, for both the ankle and the wrist speed profiles.

**Table 2:** **Goodness of fit for each model and each direction, as the percent error for the area under the fitted speed profile and the measured speed profile**.

Model	Mean (SD)% error IE inbound (*N* = 349)	Mean (SD)% error IE outbound to east (*N* = 85)	Mean (SD)% error IE outbound to west (*N* = 148)	Mean (SD)% error DP inbound (*N* = 430)	Mean (SD)% error DP outbound to north (*N* = 83)	Mean (SD)% error DP outbound to south *(N* = 291)
Sigdiscont	6.31 (2.83)	5.24 (2.61)	5.63 (2.34)	4.93 (2.14)	7.29 (2.95)	4.40 (1.78)
MMMasym	7.58 (3.17)	5.50 (3.29)	6.59 (2.83)	5.79 (2.88)	8.69 (4.21)	4.90 (1.93)
Asymgauss	8.40 (4.22)	6.26 (2.73)	8.21 (8.99)	6.67 (4.30)	9.07 (4.07)	5.92 (1.82)
LGNB	8.83 (8.67)	8.28 (9.50)	8.10 (7.60)	7.10 (6.85)	13.87 (11.86)	6.10 (6.75)
Sigcont	9.22 (3.93)	8.07 (4.72)	8.74 (4.93)	7.90 (3.39)	11.43 (4.90)	6.73 (2.23)
Gamma	10.99 (5.14)	8.65 (4.36)	10.25 (4.80)	9.27 (5.72)	12.34 (5.60)	7.99 (6.12)
GG	12.03 (6.86)	9.84 (5.21)	10.90 (5.74)	10.43 (5.49)	17.67 (11.56)	8.73 (3.44)
Beta	11.33 (16.50)	10.84 (19.10)	13.76 (24.55)	12.23 (20.67)	14.02 (15.35)	6.69 (12.25)
Symgauss	12.02 (4.68)	10.26 (4.77)	11.13 (5.30)	10.86 (5.24)	16.79 (6.98)	9.35 (3.24)
Minsnap	11.95 (5.39)	10.25 (6.26)	11.46 (6.97)	10.89 (6.27)	18.22 (7.47)	9.19 (4.06)
Minjerk	12.15 (5.66)	10.54 (6.82)	11.92 (7.40)	11.25 (6.60)	19.33 (7.57)	9.44 (4.51)
Edhol	12.18 (12.11)	13.24 (18.50)	10.61 (7.64)	12.1 (13.38)	16.05 (8.45)	9.64 (13.22)
Expo	12.49 (4.14)	11.93 (3.81)	12.54 (4.53)	11.91 (4.01)	15.28 (4.30)	10.63 (2.74)
LGN	15.66 (10.66)	11.63 (10.50)	13.45 (9.05)	12.32 (10.13)	13.74 (9.27)	10.96 (9.12)
GGgen	15.94 (21.25)	11.97 (18.05)	13.52 (16.85)	11.8 (16.16)	19.37 (23.38)	11.89 (19.07)
MMMsym	16.19 (13.24)	11.04 (8.73)	14.59 (14.25)	15.44 (15.87)	24.36 (16.40)	12.29 (12.56)
Weibull	16.84 (10.93)	16.64 (11.30)	15.74 (10.74)	15.71 (9.23)	19.78 (7.73)	14.62 (8.78)
Morasso	15.56 (7.02)	16.06 (7.55)	17.04 (9.29)	16.36 (8.19)	25.10 (8.27)	14.84 (6.93)
Biexpo	17.04 (3.26)	16.04 (2.57)	16.22 (2.72)	16.88 (2.82)	18.48 (4.03)	16.35 (2.45)

**Figure 5:**
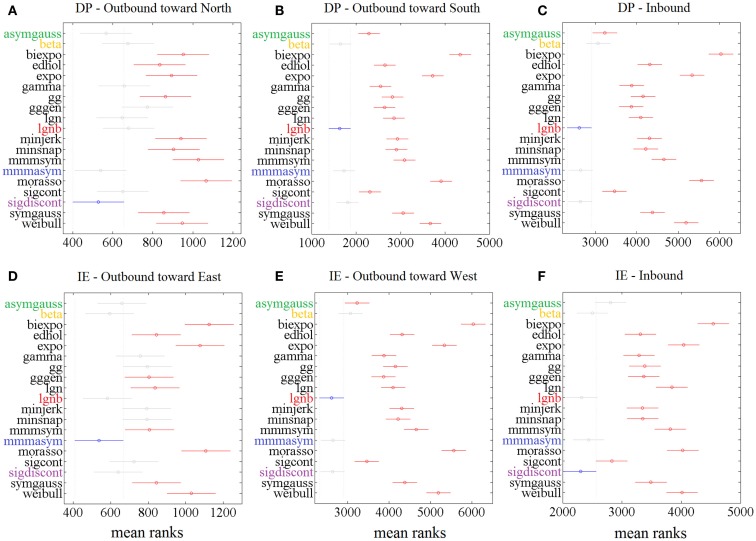
**Results of Kruskal–Wallis 1-way ANOVA and multiple comparisons’ test on AIC for different models for 1386 speed profiles**. For each movement direction, the best model (smaller mean rank AIC value) is shown in blue. Other models’ mean ranks are in red if statistically significantly different from it to 5% significance level and in gray if not. The 5 models with consistent presence in the significantly better group of models are colored. Speed profiles were taken from **(A)** 83 outbound toward north speed profiles, **(B)** 291 outbound toward south speed profiles, **(C)** 430 DP inbound speed profiles, **(D)** 85 outbound toward east speed profiles, **(E)** 148 outbound toward west speed profiles, **(F)** 349 IE inbound speed profiles.

The statistical analysis, based on the AIC, for each ankle movement separately is shown in Figure 5. The models with lower mean ranks in the Kruskal–Wallis one-way ANOVA performed better. The best performers proposed by the AIC analysis were the same as the ones proposed in Figure 3 (“Top-5” plots) and Figure 4 (“Score-18” plots): the lgnb, mmmasym, asymgauss, beta, and sigmoidal discontinuous models were consistently among the best performers and were statistically better than the rest of the models for the four out of six ankle directions. Note that for the remaining two movements (DP outbound toward north and IE outbound toward east), a few other models were statistically equivalent to the best performers, most of them marginally. This might be due to the much smaller number of overall movements for these directions, compared to all the other movements.

## Discussion

### Kinematic analysis of ankle speed profiles

In this study, we explored the speed profiles of visually evoked, visually guided, and target-directed ankle pointing movements in dorsiflexion and inversion/eversion directions. We examined the DP and IE directions because our robots train ankle movements by providing independent, active assistance in these two degrees-of-freedom, both in adults with stroke (Forrester et al., 2011, 2013, 2014; Roy et al., 2011) and children with CP (Michmizos and Krebs, 2012a). Preliminary results suggest that a focus on ankle sensorimotor control may provide a valuable contribution to locomotor therapies (Forrester et al., 2011, 2013, 2014). Our experimental setup was identical to the setup used in our clinical trials, in which the subjects are in a seated position with the weight of the anklebot being supported by the chair through a bolt (see Figure 1A). Although the anklebot is a low-friction, back-drivable robotic device with intrinsically low mechanical impedance that virtually gets out of the way during a normal movement, we still need to address whether the recorded ankle kinematics could be altered by the added inertia and friction of the robot. Preliminary studies with the anklebot examined how the device influences, among other gait parameters, the LE joint kinematics in both adults and children with LE impairments (Khanna et al., 2010; Rossi et al., 2013). None of these studies found significant changes in the spatiotemporal gait parameters in overground walking or on a treadmill. These studies, alongside the recruitment of healthy young subjects that tolerate any inertia and friction better than the impaired subjects and the support of the Anklebot’s weight from the chair, further confirm that the anklebot could not alter the ankle kinematics during the reported tasks.

Another factor of our kinematic analysis that needs to be considered is the effect of gravity on the speed profiles in DP direction. During the experiment, the weight of the Anklebot was supported by the chair; only the foot was controlled by the subject’s muscles [see Table 1 in Michmizos and Krebs (2014b)] for a complete list of these muscles). Therefore, the weight of the foot should be considered negligible, compared to the weight of the body that these muscles can support when controlling the ankle in upright position. Indeed, if gravity was a dominant factor in DP movements, it should equally affect all movements that went against gravity. However, the speed profiles for the outbound to north movements and the inbound from south movements were significantly different (Figure 2; Table 1). The same holds for the outbound to south and the inbound from north movements; their speed profiles were also significantly different. Therefore, gravity does not have a major role in the analyzed movements.

In our setup, the average peak ankle speed was above 1.5 rad/s for most of the ankle directions. The recorded peak speed was considerably lower than in the majority of previous studies that reported a peak ankle speed during the stance period of walking equal to 3.6 ± 0.2 rad/s (Winter, 1983, 2009). The relatively low peak speed of ankle movements in the present study is likely to be reflective of the differences between our experimental setup and the setup that was consistently used in all other studies (Carson et al., 2001; Kitaoka et al., 2006; Jenkyn and Nicol, 2007; Legault-Moore et al., 2012). Specifically, the other studies report ankle angles and velocities from subjects walking at different speeds on a treadmill or overground. Therefore, our setup differs from the other studies in three fundamental aspects. First, during walking, other muscles are involved in the ankle movement; for example, the TA (the largest dorsiflexor muscle) contributes less than half of the maximum voluntary torque when the ankle joint is in the midposition phase of the gait cycle, with the remainder torque presumably being provided by the long extensors of the toes (Marsh et al., 1981). On the contrary, the DP and IE movements in our experiment were controlled exclusively by the ankle muscles. Second, in the other studies, the ankle dynamics and the stiffness of the muscles that support the ankle change within each gait cycle and also with walking speed (Au et al., 2006; Kim and Park, 2011; Lee et al., 2011, 2014). The ankle stiffness, in its turn, affects significantly the ankle torque in the absence of fatigue (McNair et al., 2002). Third, the tested ankle ROM in our study (0.2 rad; 12°) was lower than the maximum ROM in walking, which can be equal to 35° (for DP) in high-speed walking (Novacheck, 1998). With the rise time to peak tension in the ankle muscles being relatively constant regardless of the speed (Perrine and Edgerton, 1977), the 0.2-rad ankle angle was not sufficiently distal in the arc of motion to allow for rise time to peak tension in the muscles. In support of our argument, Palmer reports a peak speed of 2 rad/s for a dorsiflexion of 0.2 rad and a peak speed of 5 rad/s for a dorsiflexion of 0.35 rad during walking; see Figure 2.2 in Palmer (2002).

Skewness and kurtosis analyses showed that the larger proportion of data was asymmetric. Consistently, asymmetric models of speed profiles performed better than symmetric ones. Overall kurtosis values were less than those expected for symmetric Gaussian curves, i.e., speed profiles mostly appeared to be platykurtic, with smaller tails and wider peaks. These results were in agreement with the kinematic analysis of the wrist speed profiles (Vaisman et al., 2013) and further spurred our comparisons across UE and LE modalities.

### Speed profile modeling and generalization of models across modalities

Under the general assumption that models are hardly ever true but some of them fit the data well enough to allow for useful inference, we sought to find how our modeling analysis compared with our and others’ studies on different modalities. The models that we employed here were pulled from a set of models that were initially designed to describe UE movements with various degrees of complexity, including reaching, drawing, handwriting, and wrist movements. In terms of the neurophysiological assumptions implied, our models could be categorized into two main categories: the kinematics-oriented and the dynamics-based models. The kinematics-oriented models hypothesize that the CNS controls the trajectory of the joint in space by possibly minimizing the error at the end effector without controlling the actual joint or the muscles. Such models are the Morasso model (Morasso and Ivaldi, 1982) and its modified versions (Maarse, 1987), the minimum jerk (Flash and Hogan, 1985), the minimum snap (Edelman and Flash, 1987), the Gutman and Gottlieb (gg) (Gutman and Gottlieb, 1991), the Gaussian, lgn and lgnb (Plamondon and Alimi, 1997; Plamondon et al., 2003), and the beta, gamma, and Weibull models (Plamondon et al., 1993, 2003). Diametrically opposed, the dynamics-based models assume that trajectory formation mechanisms are directly related to the geometry and mechanical properties of muscles, which can be seen as generators of force, oscillation, or speed (Plamondon et al., 1993). Such models are the Eden-Hollerbach (Hollerbach, 1981), and Plamondon and Lamarche (1986) models. To fully comply with our wrist modeling study, we also included the biexponential model (Stein et al., 1988). For a thorough presentation of the models, see Vaisman et al. (2013).

The comparison of the model performances between our LE study that focused on the ankle joints and other UE studies that focused on the hand, the arm, or the wrist (Plamondon et al., 1993; Vaisman et al., 2013) could expand our insight on how the brain controls movement and recovers from injury. The latter should be enlightened by the fact that the LE movements, being older phylogenetically, are not necessarily controlled in the same fashion as the UE movements. For example, although both wrist and ankle are pivot joints, they differ in function. The wrist joint, being an ellipsoid joint, permits movements around two axes, flexion and extension around the transverse axis, and adduction and abduction around the antero-posterior, whereas the ankle joint, being a hinge joint, permits movement in one plane around a transverse axis (Rogers, 2010). Despite the difference in the rotational degrees-of-freedom between the two joints, alongside other differences (in limbs, functional role, etc.), the similarity between the two modeling results is remarkable: Not only do the two joints share the top-performing model (lgnb) but also the other models in the top-5 list (mmmasym, asymgauss, beta, and sigdiscont) and even the models’ order in the list are the same; compare Figure 3 in this paper with Figure 4 in Vaisman et al. (2013). The two joints’ speed profiles shared, as well, the worst-performing models (expo, weibul, moraso, and biexpo). Based on these results, it seems reasonable to assume that a single model describes, equally well the rotational movements in the two distinct pivot joints. Our results are in further agreement with other UE (drawing) studies that find that the lgnb model outperforms all other models, while the asymgauss and the sigdiscont models are validated among the top performers. In agreement with our study, the models found to perform poorly in handwriting are the morasso, biexpo, and expo models (Plamondon et al., 1993). The observation that asymmetric models perform better than the symmetric ones is not only consistent with our modeling work on the wrist movements but also in agreement with prior work that showed that single-joint movements often have asymmetric speed profiles (Nagasaki, 1989; Wiegner and Wierzbicka, 1992). A plausible neurophysiological explanation for the asymmetry lies on either muscle viscosity or patterns of muscle activation (Jaric et al., 1998).

Among the top-5 best performing models, the lgnb and the beta function are of particular value as they are continuous models. A model that is discontinuous at the peak of the speed profile has infinite acceleration at that point, which is helpful in reproducing accurately the handwriting dynamics but it is not realistic for ankle or wrist pointing movements. Discontinuous models are, among others, the sigdiscont, the mmmasym, and the asymgauss that were found in the top-5 performers. In addition to being a continuous model, our best-fit model, the lgnb function is neurophysiologically plausible and has high flexibility in the description of both positively and negatively skewed ankle speed profiles (see Figure 2) (Plamondon and Alimi, 1997; Plamondon et al., 2003). The lgnb has also been used successfully to model drawing movements of unimpaired subjects (Plamondon et al., 1993), and reaching and drawing movements of subjects recovering from stroke (Rohrer et al., 2002; Dipietro et al., 2009). The top performance of the beta model, for both ankle and wrist movements, is consistent with our previous work where the beta function was successfully used to model speed profiles of submovements in shoulder and elbow movements of recovering stroke patients (Krebs et al., 1999).

However, any generalization of our results across modalities should be made with care. Other voluntary movements exist that cannot be simulated with the models that we used here. For example, eye movements (e.g., saccades, conjugate, and vergence pursuit) are arguably the most frequent of all voluntary movements. Although both pursuit and saccades are handled by the same neural circuitry (Galiana and Outerbridge, 1984; Galiana, 1991), they serve different functions: saccadic eye movements exploit the foveated structure of the eye, quickly reorienting it to place a newly selected or fleeing target near the fovea, while pursuit movements allow the eyes to closely follow a moving object up to a certain speed. The speed profile of saccadic eye movements is more leptokurtic than the bell-shaped speed curves that we described. Importantly, we cannot control the velocity of a saccade or its duration, which are solely determined by the saccade amplitude (Wolfgang, 1991). An important difference, though, between saccades and movements that we analyzed here is that saccades are blind eye movements; during saccade, vision is not used in a negative feedback loop as in pursuit (Robinson, 1965, 1968). Therefore, we speculate that the speed profiles we have seen here and in our previous studies are the results of a visual-motor integration that might generalize well across visually guided voluntary movements.

### Evidence that lower and upper extremity discrete movements are controlled by a single invariant strategy

The main finding of our study is that the tested speed components of the visually guided, goal-directed discrete movements remain invariant across LE and UE. This similarity across modalities suggests that an effective therapeutic intervention for the LE should follow the current knowledge of how the CNS controls and learns UE movement (Michmizos and Krebs, 2012b). Motor control theories have predominantly focused on UE where research on practicing target-directed movements has revealed the large capacity of the motor system to learn (Schmidt and Wrisberg, 2004). Studies frequently assume but rarely examine whether LE movements obey the same principles that underlie motor learning in UE or how LE speed profiles could be seen under the scope of motor control theories.

We examined our results in light of the two most prevalent motor control theories, namely the motor program theory and the dynamical systems theory. According to the motor program theory, motor instructions are specified by the CNS where a motor program organizes, initiates, and carries out intended actions, based on sensory stimuli or central processes (Schmidt and Lee, 1988). The motor programs are hardwired and stereotyped neural connections and include central pattern generators (CPGs), which are networks of interneurons capable of generating bilateral rhythmic movements – such as swimming or walking – in the absence of descending and sensory inputs (Ijspeert, 2008). Although sensory inputs are not required to produce a movement, they are important in adapting and modulating the movement. According to the dynamic systems theory, motor instructions are influenced by the environment and the interaction among the body, the limbs, and the nervous system (Bernstein, 1967). A movement pattern self-organizes as a function of the ever-changing constraints placed upon it (Kamm et al., 1990). Each of these subsystems, that has the potential to change, is referred to as control parameter and may be the target of therapeutic intervention to improve motor learning.

Both motor control theories have integrated concepts from the ecological theory of motor control (Gibson, 1966) into their constructs and, therefore, have influenced a task-oriented therapeutic approach, i.e., an approach that includes meaningful activities within the patient’s natural environment. Our “assist-as-needed” robotic therapists for both the UE and the LE align with this approach in terms of providing intervention within the context of the individual’s preferences and needs. As a motor function, however, gait is unique in human beings who are the only exclusively bipedal mammals. A view that has received wide support is that the control of locomotion is achieved through an interplay of CPGs and sensory influences (Van de Crommert et al., 1998). Whether LE movement problems are caused by abnormal CPGs, higher level motor programs or the interconnection between them remain to be proven.

The underlying invariant features in the visually guided discrete movements across lower and upper limbs suggest that the CNS may control these movements by eliciting a stereotypical motor program that consists of a prestructured set of motor commands. Such commands could be constructed at the highest cortical levels and then conveyed to the lowest centers in the hierarchy responsible for executing the movement. Several invariant features in UE movements, such as smooth trajectories with uni-modal speed profiles, may also hold for the LE. Another strong indication for an invariant motor signature among UE and LE comes from our recent study on the ankle RT: as a task increases in complexity, the amount of time needed to organize the motor program would increase. Indeed, the RT in the ankle increases with the number of potential stimuli, as would be predicted by the Hick-Hyman law that has long been observed in the UE (Michmizos and Krebs, 2014a).

In our task-based rehabilitation studies, recovery is found to be best produced by practicing purposeful, goal-oriented tasks (Hogan et al., 2006). Finding an invariant motor strategy across modalities enables us to explore the expansion of the task goals that we have used for the UE to the LE (Michmizos and Krebs, 2012a). This will allow us to structure the physical environment by allowing choices of movement solutions and manipulating performance, environmental, or task variables. Stepping away from the training of repetitive rhythmic LE movements, this work aims to inspire approaches that include meaningful LE activities within the individual’s natural environment. In conclusion, this work shows alternatives to help patients relearn the correct actions and further support the goal-directed (activity-based) training of the LE.

### Implications for LE robotic therapy

Historically, LE robotics for neurological diseases tried to impose rhythmic patterns of whole-body movements. However, the evaluation of such devices that mimic the kinematics of rhythmic leg movements during BWS treadmill training yielded poor results (Duncan et al., 2011; Dobkin and Duncan, 2012). Targeting individual joints and going beyond exclusive rhythmic training is a different strategy for LE robotics that has already shown promising results. Recent studies using the Anklebot suggest that a focus on ankle discrete movements provides a valuable contribution to locomotor therapies (Forrester et al., 2011, 2013, 2014). In addition, our lab has recently introduced a new LE manipulandum that enables the training of discrete and rhythmic movements as well as balance (Susko and Krebs, 2014). The neurophysiological basis of our work is the model of dynamic primitives (Hogan and Sternad, 2012), according to which the sensorimotor control could be broken down into three elemental primitives: submovements, oscillations, and mechanical impedances. Just like infants learning to walk, severe stroke survivors use discrete steps. Discrete movements have also been observed in UE reaching movements, hypothesized to be composed of a superposition of submovements. Since rhythmic and discrete movements are known to be controlled by different neural mechanisms (Schaal et al., 2004; Hogan and Sternad, 2007), LE rehabilitation needs to address both movements.

Up to now, there is a shortage of studies on the neurophysiological mechanisms underlying the ankle sensorimotor control and trajectory formation. However, similar to previous studies, we simulated ankle trajectories with the point of interest fixed on their performance in reproducing speed profiles of simple, fast target-directed movements (Plamondon et al., 1993; Vaisman et al., 2013). This focus was consistent with our goal on verifying whether the existent UE models are sufficient to describe the ankle speed profiles or whether we needed to develop new models of LE movements. Our model validation confirms the adequacy of some of the UE models. These results support embedding such models into the design of ankle robotic tools for neurorehabilitation and using them to inform quantitatively the sensorimotor recovery in neurological diseases that originate in the brain but affect the periphery.

Much current robotic research on neurorehabilitation focuses on adapting the behavioral intervention to each patient’s special needs and abilities. The most prominent working hypothesis is that the processes that underlie motor rehabilitation are similar to the processes that underlie motor learning (Hogan et al., 2006). In terms of kinematics, to quantify motor learning, one needs to assess the level of deviation from the ideal trajectory. Although other, more sophisticated methods exist that quantify the electrical and biochemical changes in the brain, as it undergoes plasticity, the brain is an intrinsically complex system and any data acquired from it, especially during a therapy session, are hard to interpret and, therefore, to exploit in any treatment. The most robust measure of quantifying motor learning, up to now, is through behavioral changes (Schmidt and Lee, 1988), conveniently assessed from the kinematics from the robot itself.

Motor recovery follows an exponential progression similar to a motor learning “law of practice” or, especially in complex tasks, a two-time scale exponential function (Newell et al., 2009). The theory of multiple time scales is further supported by neurophysiological studies (Karni et al., 1998; Bernacchia et al., 2011). According to this theory, the two superimposed exponential functions represent adaptation and learning processes. One characteristic time scale is relatively fast and captures the rapid adaptive change (warm-up) in performance at the beginning of a practice session. The other time scale is relatively slow and captures the persistent change that is more typically associated with learning. But scoping the learning curves associated with the performance metrics is not the only direct implication of our modeling results. This work has a great potential to enhance rehabilitation efforts in any population with LE deficits by, for example, assessing the level of motor impairment and improvement. Knowledge of the best-fit models on ankle pointing movements enhances the ability to interpret both individual differences (pre- and post-intervention) and group differences (between pathological and non-pathological individuals) during kinematic investigations.

## Conclusion

The incorporation of behavior quantification techniques to LE rehabilitation using a robotic device seems very promising. The robot becomes a platform that combines neuroscience, modeling, and physical therapy among other disciplines. The results presented here could be interpreted under both an engineering and a scientific perspective. The modeling of ankle kinematics will enable us to engineer therapeutic devices that first identify the ability of the patient to move and point with the ankle, and then independently adjust the therapeutic parameters and the number of its repetitions. Scientifically, the comparison of our modeling results with other UE studies build the support for an invariant theory of sensorimotor control of discrete movements across upper and lower limbs. This interpretation of our results, in light of the contemporary motor control theories, emphasizes the importance of individual therapeutic tasks that incorporate environmental movement constraints. Models such as the ones we used here could be used to compare distinct neurologic rehabilitation approaches with respect to assumptions underlying normal and abnormal movement control and recovery of function. However, the right training dosage of discrete and rhythmic movements as well balances to maximize recovery outcomes and how to tailor the training to a particular patient’s needs remain to be verified.

## Conflict of Interest Statement

The authors declare that the research was conducted in the absence of any commercial or financial relationships that could be construed as a potential conflict of interest.

## Supplementary Material

The Supplementary Material for this article can be found online at http://www.frontiersin.org/Journal/10.3389/fnhum.2014.00962/abstract
