# Supplementary material for: A Comparative Analysis of Speed Profile Models for Ankle Pointing Movements: Evidence that Lower and Upper Extremity Discrete Movements are Controlled by a Single Invariant Strategy
Source: Front Hum Neurosci. 2014 Nov 27;8:962. doi: 10.3389/fnhum.2014.00962 (PMC4245889; doi:10.3389/fnhum.2014.00962)
Supplement: Supplementary file 1 [file Data_Sheet1.PDF]

1. Appendix A

Geometrical characteristics of the foot-robot kinematics

Displacements of the linear actuators were measured by linear encoders. Ankle angles for DP and IE movement were estimated using a simple linearized mathematical model of the shank-ankle-foot system. The ankle angles measured from the neutral position in the DP plane, θ_{dp}, and IE plane, , θ_{IE}, were estimated as follows:

$$\theta_{dp} = \sin^{-1}(x) + \theta_{dp,offset} \tag{1}$$

$$\theta_{ie} = \tan^{-1}\left(\frac{x_{right} - x_{left}}{x_{tr,width}}\right) + \theta_{ie,offset} \tag{2}$$

where

$$x = \left(\frac{x_{tr,len}^2 + L_{shank}^2 - x_{link,disp}^2}{2x_{tr,len}L_{shank}}\right), \tag{2}$$

$$x_{link,disp} = \left(\frac{x_{av-act,len} - x_{right}}{2}\right) + \left(\frac{x_{av-act,len} - x_{left}}{2}\right), \tag{3}$$

$\theta_{dp,offset}$ was the offset in the DP ankle angle, $\theta_{ie,offset}$ was the IE offset angle, x_{right} and x_{left} were the lengths of the right and left actuators, respectively, $x_{tr,width}$ was the transverse “ball-to-ball” width, $x_{tr,len}$ was the distance between the line of action of actuator force and the point of attachment between the ankle and the robot in the sagittal plane, L_{shank} was the shank length, $x_{link,disp}$was the linear displacement of the linkage, and $x_{av-act,len}$ was the average actuator length.

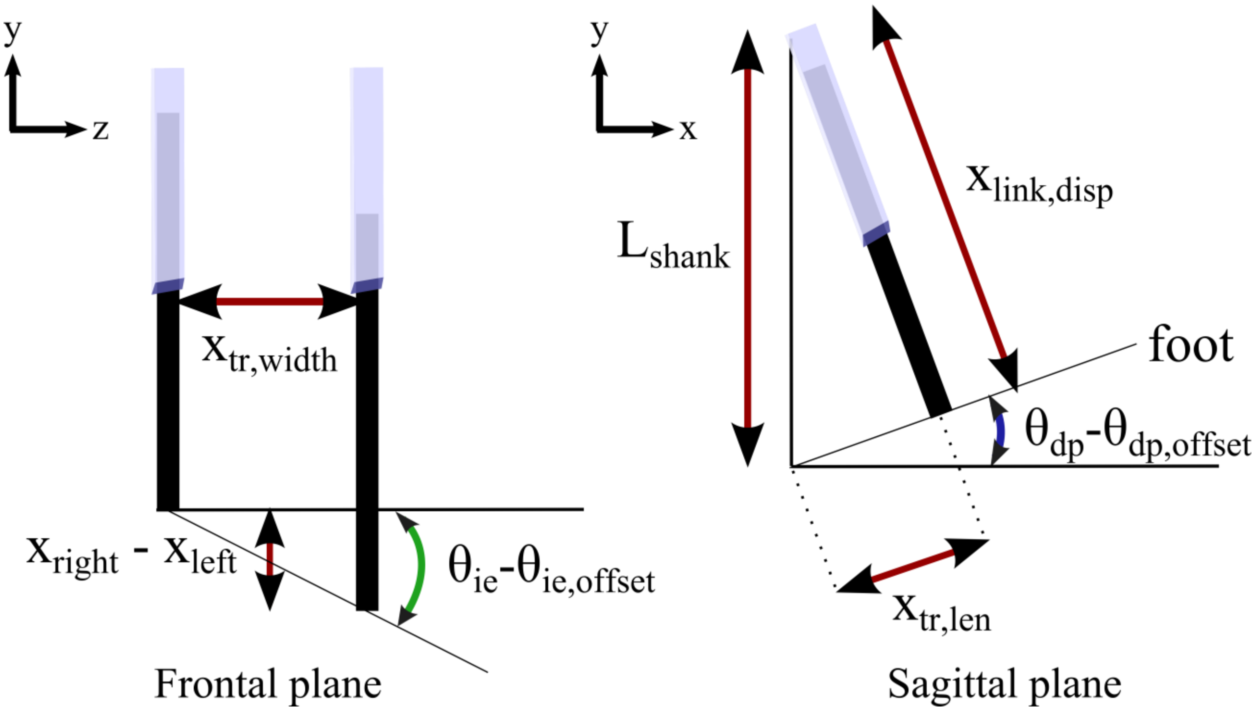

Figure S1 Schematic of the Anklebot and the geometrical relationships among the parameters used to estimate ankle angles.

2. Appendix B

Model equations

For each model with parameters $P_1 \ldots P_n$, t_0 was the starting time of the movement, t_1 was the stopping time for the movement and t_m was the time when maximum velocity occurred. Abbreviations used in the plots and initial conditions are also shown.

Eden and Hollerbach Model (edhol) (Hollerbach, 1981;Plamondon et al., 1993) (P_1=1, P_2=1, P_3=0.5, P_4=1, P_5=4, P_6=3, P_7=0)

$$V = \begin{cases} \sqrt{\{P_1 \sin[P_2(t-P_3)] + P_4\}^2 + \{P_5 \sin[P_6(t-P_7)]\}^2} & t_0 \le t \le t_1 \\ 0 & elsewhere \end{cases}$$

Minimum Jerk Model (minjerk) (Plamondon et al., 1993) (P_1=10, P_2=0, P_3=1)

$$V = \begin{cases} P_1(t-P_2)^2(t-P_3)^2 & t_0 \le t \le t_1 \\ 0 & elsewhere \end{cases}$$

Minimum Snap Model (minsnap) (Edelman and Flash, 1987;Plamondon et al., 1993) (P_1= -1, P_2=0, P_3=1)

$$V = \begin{cases} P_1(t-P_2)^3(t-P_3)^3 & t_0 \le t \le t_1 \\ 0 & elsewhere \end{cases}$$

Morasso and Mussa-Ivaldi Minimum Acceleration Model (morasso) (Morasso and Ivaldi, 1982;Plamondon et al., 1993) (P_1=-10, P_2=0, P_3=1)

$$V = \begin{matrix} P_1(t-P_2)(t-P_3) & t_0 \le t \le t_1 \\ 0 & elsewhere \end{matrix}$$

Plamondon and Lamarche Model (expo) (Plamondon et al., 1993) (P_1=20, P_2=0.5, P_3=0, P_4=9, P_5=5, P_6=1)

$$V = \begin{cases} P_1 e^{-P_2(t-P_3)} & t_0 \le t \le t_m \\ P_4 e^{-P_5(t-P_6)} & t_m \le t \le t_1 \\ 0 & elsewhere \end{cases}$$

Biexponential Model (biexpo) (Stein et al., 1988) (P_1=0.5, P_2=0.5, P_3=0.5)

$$V = \begin{cases} P_1 e^{-|t-P_2|/P_3} & t_0 \le t \le t_1 \\ 0 & elsewhere \end{cases}$$

Plamondon Lognormal Model (lgn) (Plamondon et al., 1993) (P_1=1, P_2=-1, P_3=10, P_4=1)

$$V = \begin{cases} \dfrac{P_1}{t-P_2} e^{-P_3[\ln(t-P_2)-P_4]^2} & t_0 \le t \le t_1 \\ 0 & elsewhere \end{cases}$$

Plamondon Lognormal Model with Support Bound (lgnb) (Plamondon et al., 1993) (P_1=5, P_2=-1.5, P_3=1.5, P_4=2, P_5=0.5)

$$V = \begin{cases} \dfrac{P_1}{(t-P_2)(P_3-t)} e^{-P_4[\ln((t-P_2)/(P_3-t))-P_5]^2} & t_0 \le t \le t_1 \\ 0 & elsewhere \end{cases}$$

Beta Function Model (beta) (Plamondon et al., 1993) (P_1=2, P_2=-1, P_3=8, P_4=2, P_5=7)

$$V = \begin{cases} P_1(t-P_2)^{P_3}(P_4-t)^{P_5} & t_0 \le t \le t_1 \\ 0 & elsewhere \end{cases}$$

Gamma Function Model (gamma) (Plamondon et al., 1993) (P_1=0.4, P_2=-0.05, P_3=5, P_4=8)

$$V = \begin{cases} P_1[P_4(t-P_2)]^{P_3} e^{-P_4(t-P_2)} & t_0 \le t \le t_1 \\ 0 & elsewhere \end{cases}$$

Weibull Model (weibull) (Plamondon et al., 1993) (P_1=1, P_2=-1, P_3=2)

$$V = \begin{cases} P_1[(t-P_2)]^{P_3-1} e^{-(t-P_2)^{P_3}} & t_0 \le t \le t_1 \\ 0 & elsewhere \end{cases}$$

Sigmoidal Continuous Model (sigcont) (Plamondon et al., 1993) (P_1=1.1, P_2=0, P_3=2, P_4=4)

$$V = \begin{cases} P_1 \dfrac{t-P_2}{[1+P_3(t-P_2)P^4]^2} & t_0 \le t \le t_1 \\ 0 & elsewhere \end{cases}$$

Sigmoidal Discontinuous Model (sigdiscont) (Plamondon et al., 1993) (P_1=2, P_2=0, P_3=1, P_4=7, P_5=54, P_6=-0.2, P_7=1, P_8=8)

$$V = \begin{cases} P_1 \dfrac{t-P_2}{[1+P_3(t-P_2)P^4]^2} & t_0 \le t \le t_m \\ P_5 \dfrac{t-P_6}{[1+P_7(t-P_6)P^8]^2} & t_m \le t \le t_1 \\ 0 & elsewhere \end{cases}$$

Gutman and Gotlieb Original Model (gg) (Plamondon et al., 1993) (P_1=20, P_2=0, P_3=0.5)

$$V = \begin{cases} P_1(t-P_2)^2 e^{-((t-P_2)^3/P_3)} & t_0 \le t \le t_1 \\ 0 & elsewhere \end{cases}$$

Gutman and Gotlieb Generalized Model (gggen) (Plamondon et al., 1993) (P_1=20, P_2=-0.5, P_3=3, P_4=1)

$$V = \begin{cases} P_1(t-P_2)^{P_3-1} e^{-((t-P_2)^{P_3}/P_4)} & t_0 \le t \le t_1 \\ 0 & elsewhere \end{cases}$$

Symmetric Plamondon Gaussian Model (symgauss) (Stein et al., 1988;Plamondon et al., 1993) (P_1=3, P_2=0.5, P_3=0.5)

$$V = \begin{cases} P_1 e^{-((t-P_2)/P_3)^2} & t_0 \le t \le t_1 \\ 0 & elsewhere \end{cases}$$

Asymmetric Plamondon Gaussian Model (asymgauss) (Plamondon et al., 1993) (P_1=3, P_2=0.5, P_3=0.5, P_4=27, P_5=0.5, P_6=0.5)

$$V = \begin{cases} P_1 e^{-((t-P_2)/P_3)^2} & t_0 \le t \le t_m \\ P_4 e^{-((t-P_5)/P_6)^2} & t_m \le t \le t_1 \\ 0 & elsewhere \end{cases}$$

Symmetric Morasso, Mussa-Ivaldi and Maarse Model (mmmsym) (Maarse, 1987;Plamondon et al., 1993) (P_1=2, P_2=-5, P_3=-1)

$$V = \begin{cases} P_1\{1-\cos[P_2(t-P_3)]\} & t_0 \le t \le t_1 \\ 0 & elsewhere \end{cases}$$

Asymmetric Morasso, Mussa-Ivaldi and Maarse Model (mmmasym) (Plamondon et al., 1993) (P_1=1, P_2=-4, P_3=0, P_4=18, P_5=-7, P_6=-1)

$$V = \begin{cases} P_1\{1-\cos[P_2(t-P_3)]\} & t_0 \le t \le t_m \\ P_4\{1-\cos[P_5(t-P_6)]\} & t_m \le t \le t_1 \\ 0 & elsewhere \end{cases}$$
